# Supplementary material for: NSs amyloid formation is associated with the virulence of Rift Valley fever virus in mice
Source: Nat Commun. 2020 Jul 1;11:3281. doi: 10.1038/s41467-020-17101-y (PMC7329897; doi:10.1038/s41467-020-17101-y)
Supplement: Supplementary file 1 — Supplementary Information [file 41467_2020_17101_MOESM1_ESM.pdf]

NSs amyloid formation is associated with the virulence of Rift Valley fever virus in mice

Léger et al.

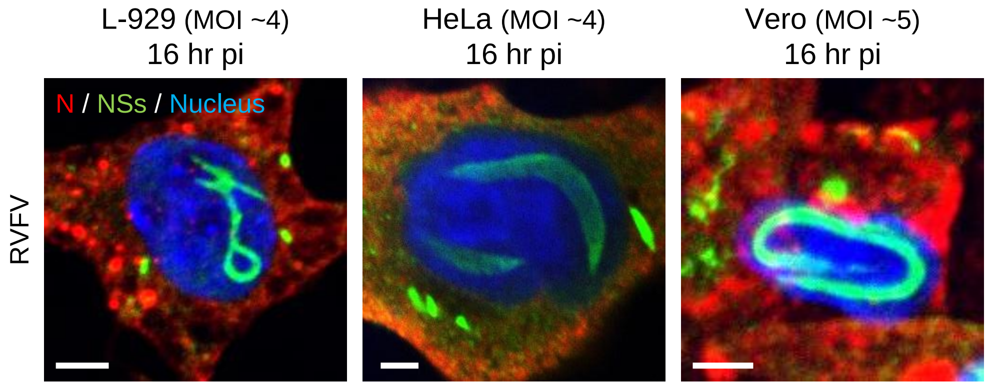

Supplementary Figure 1. NSs forms nuclear and cytosolic aggregates in L-929, HeLa, and Vero cells. L-929, HeLa, and Vero cells were infected with Rift Valley fever virus (RVFV) at indicated multiplicities of infection (MOIs) for 16 hr. Infected cells were then imaged by confocal microscopy after staining with Hoechst (blue) and antibodies (Abs) against the viral proteins NSs (green) and N (red). Images are representative of 3 independent experiments. Scale bars, 5 µm.

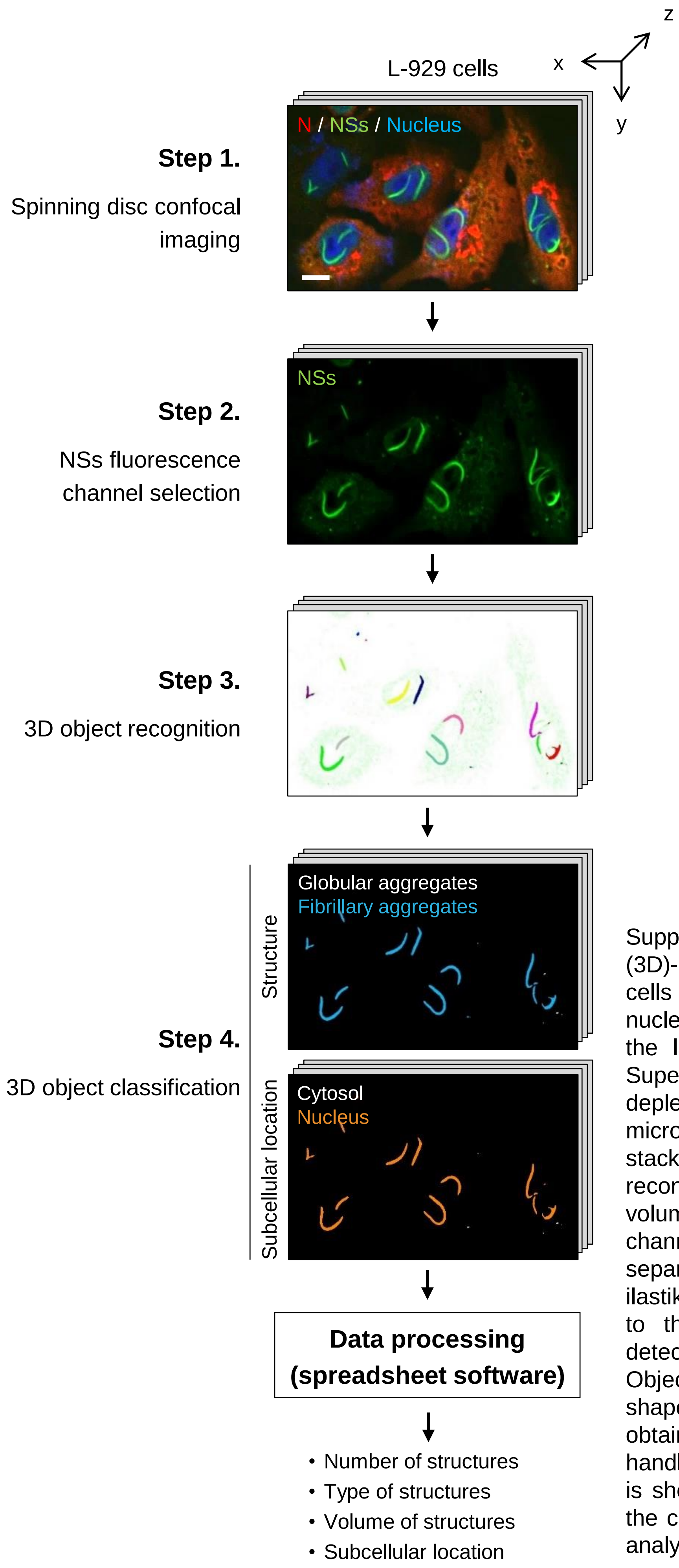

Supplementary Figure 2. Three-dimensional (3D)-computer-based image analysis. Infected cells are immunostained against the viral nucleoprotein N (red) and NSs (green) before the labeling of nuclei with Hoechst (blue). Super-resolution stimulated emission depletion (STED) or spinning disc confocal microscopy is employed to image series of Z-stack focal planes, which allows for 3D-reconstruction and the analysis of objects' volume (step 1). The fluorescence color channels are split and further processed separately (step 2). The deep learning-based ilastik software is used and algorithms applied to the series of Z-stack images for the detection and recognition of objects (step 3). Objects are next classified according to their shape and cellular location (step 4). The obtained values are exported for further handling to a spreadsheet software. Example is shown for infected L-929 cells. Of note, in the case of STED microscopy, only NSs was analyzed. Scale bar, 10 µm.

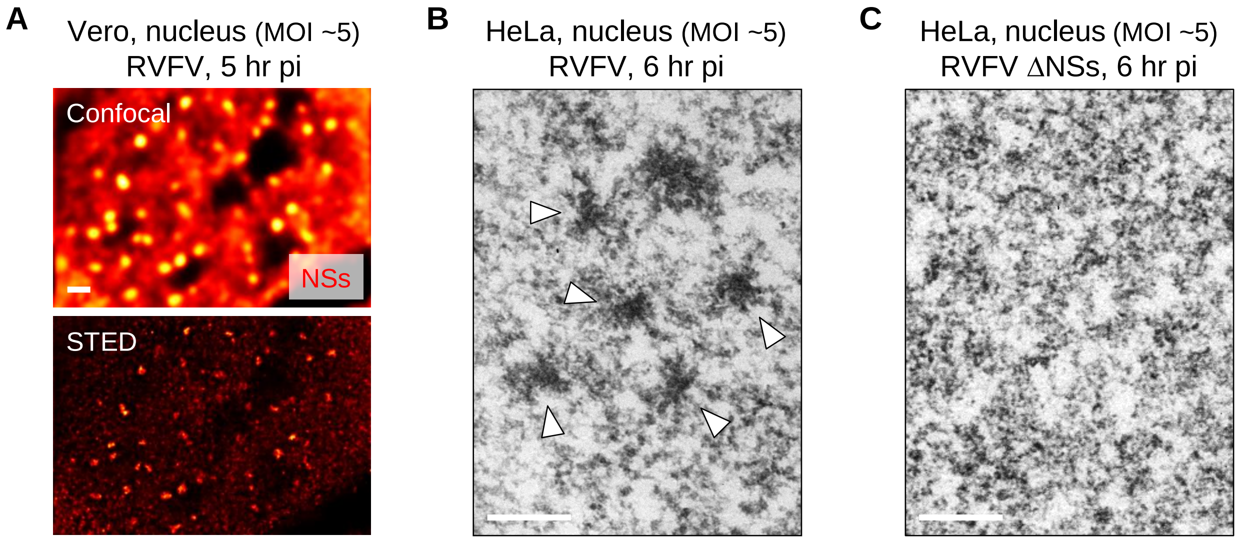

Supplementary Figure 3. Ultrastructure of nuclear bodies in RVFV-infected cells. (A) Nuclei of infected Vero cells were imaged by STED microscopy after staining with Abs against NSs 5 hr post-infection (pi). Confocal (top panel) and STED (bottom panel) images are shown. NSs appears in red/yellow. Experiments were repeated independently thrice with similar results. Scale bar, 1 µm. (B and C) Electron micrographs of thin-section HeLa cells 6 hr after infection with RVFV (B) and RVFV lacking the NSs sequence (RVFV ΔNSs) (C). White arrowheads indicate nuclear rosette-island arrangements. Images are representative of 3 independent experiments. Scale bars, 200 nm.

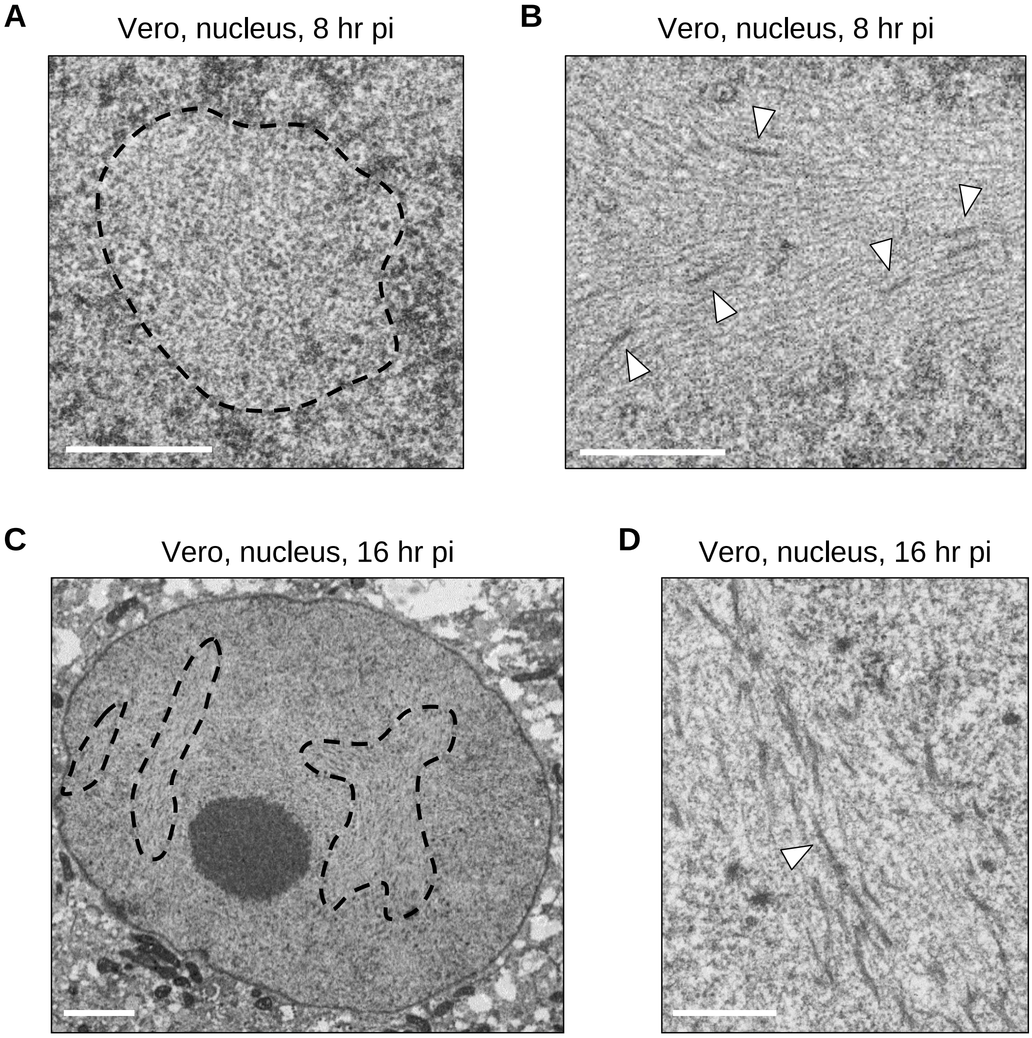

Supplementary Figure 4. NSs fibrils form denser structures at later stages of infection. (A and B) Vero cells were infected with RVFV (MOI ~5) and imaged by transmission electron microscopy (TEM) 8 hr pi. The black dashed line indicates a cross-section of one nuclear NSs fibrillary aggregate. White arrowheads indicate individual fibrils locally annealed laterally and forming electron dense structures. Images are representative of 5 independent experiments. Scale bars, 500 nm. (C and D) Vero cells were infected at a MOI of 5 for 16 hr. NSs fibrillary aggregates pervaded substantial parts of nuclei (outlined by the black dashed lines). The white arrowhead designates the typical width of the thickest regions in fibrillary aggregates. Virtually all nuclear NSs fiber-bundles were densely packed and individual fibrils very hard to depict. Experiments were repeated independently thrice with similar results. Scale bars, 500 nm.

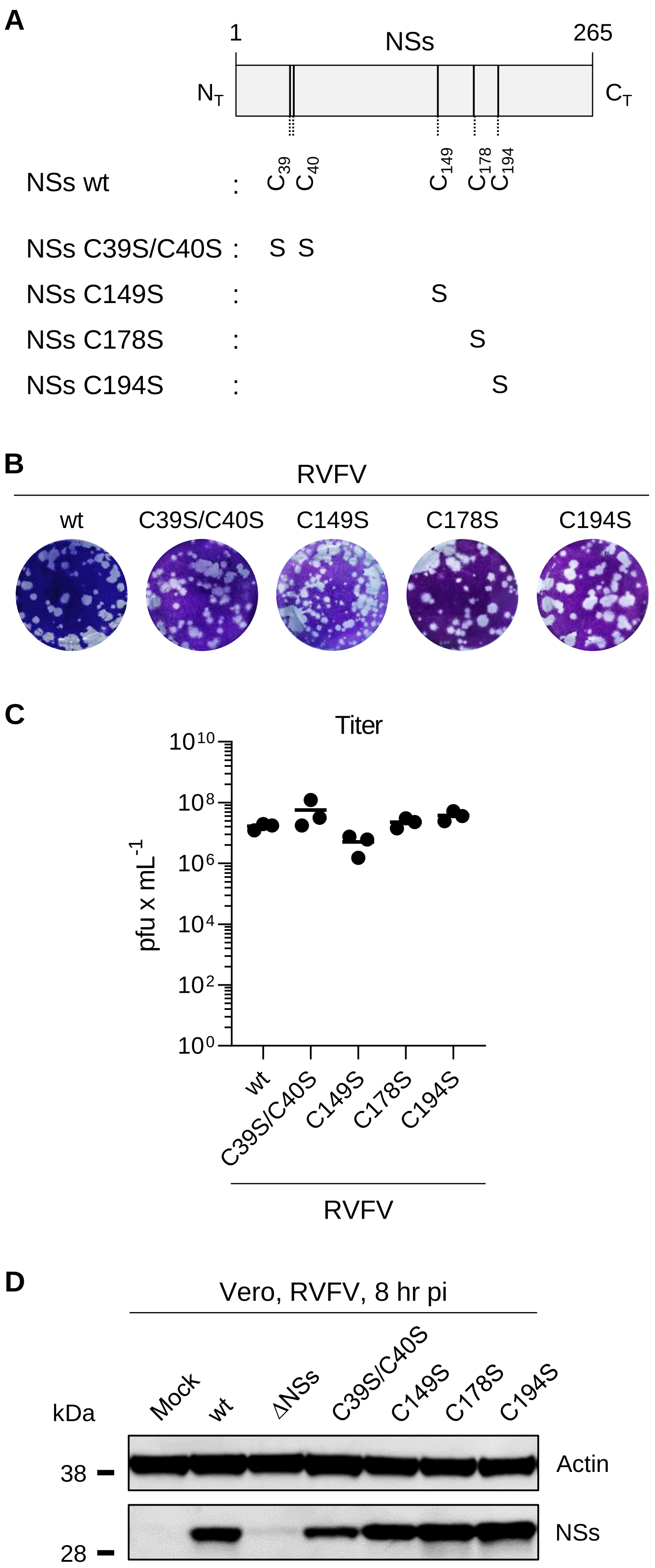

Supplementary Figure 5. Recovery and characterization of NSs cysteine mutants. (A). The five cysteine residues in the NSs sequence were each exchanged against serine and the corresponding virus mutants rescued from plasmid DNAs. (B) Plaque-forming assay used for the titration of RVFV mutants in monolayers of Vero cells. After 5 days of incubation at 37°C, plaques were colored with crystal violet. (C) Titer of the RVFV mutants after rescue and passages in Vero cells in comparison of the wild type (wt) strain. Points represent replicates (n = 3). Results are representative of two independent virus productions. Center line, mean. pfu, plaque-forming units. (D) Vero cells were exposed to RVFV and the four mutant viruses. Infected cells were lysed 8 h later, and then, analyzed by SDS-PAGE and western blotting (WB) under reducing conditions. Results are representative of 3 independent experiments.

A RVFV NSs wt (MOI ~5), Vero, 16 hr pi

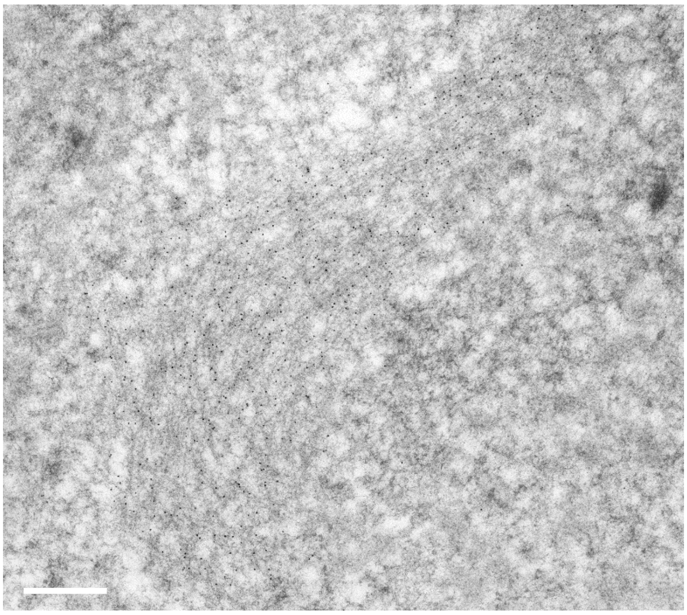

B RVFV NSs C39S/C40S (MOI ~5), Vero, 16 hr pi

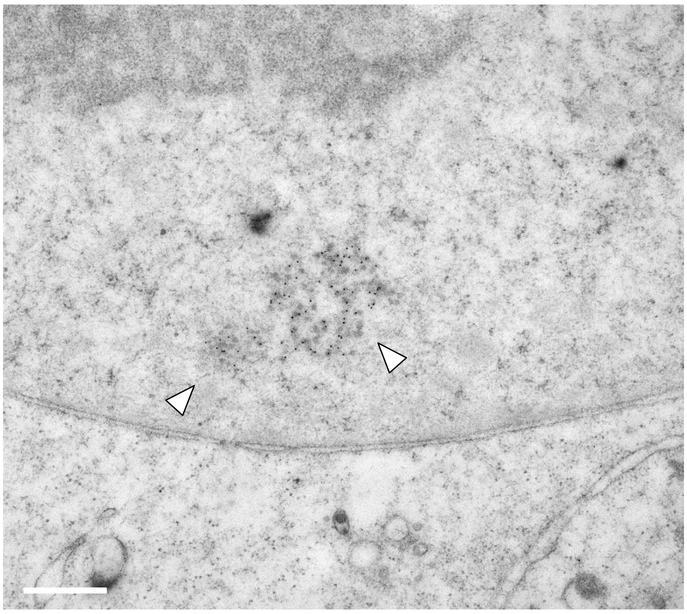

C RVFV NSs C149S (MOI ~5), Vero, 16 hr pi

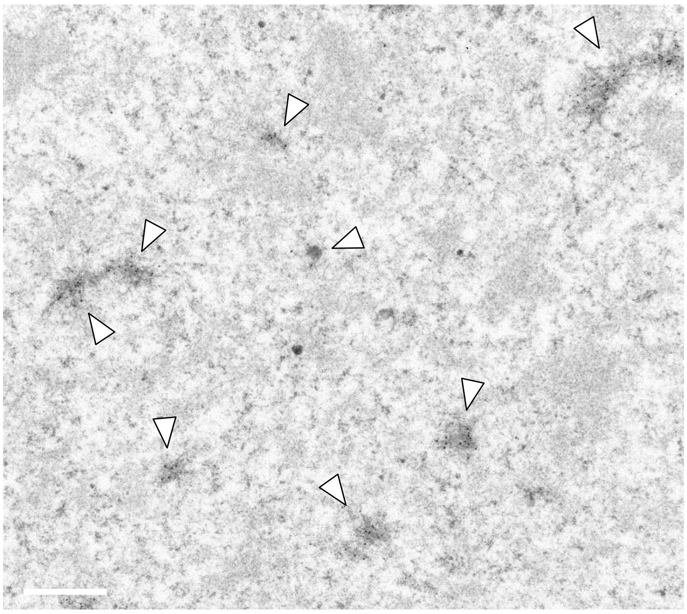

Supplementary Figure 6. Ultrastructure of RVFV NSs C39S/C40S and C149S. (A to C). Vero cells were exposed to RVFV and the two mutant viruses NSs C39S/C40S and C149S (MOI ~5) for 16 hr. Infected cells were fixed and subjected to immunogold labeling of NSs before imaging by TEM. The typical wt NSs fiber-bundles (A), the clumps built from the NSs mutant C39S/C40S (B, white arrowhead), and the short fibrils made of the NSs mutant C149S (C, white arrowhead) are seen covered by gold beads (black spots). Experiments were repeated independently thrice with similar results. Scale bars, 500 nm.

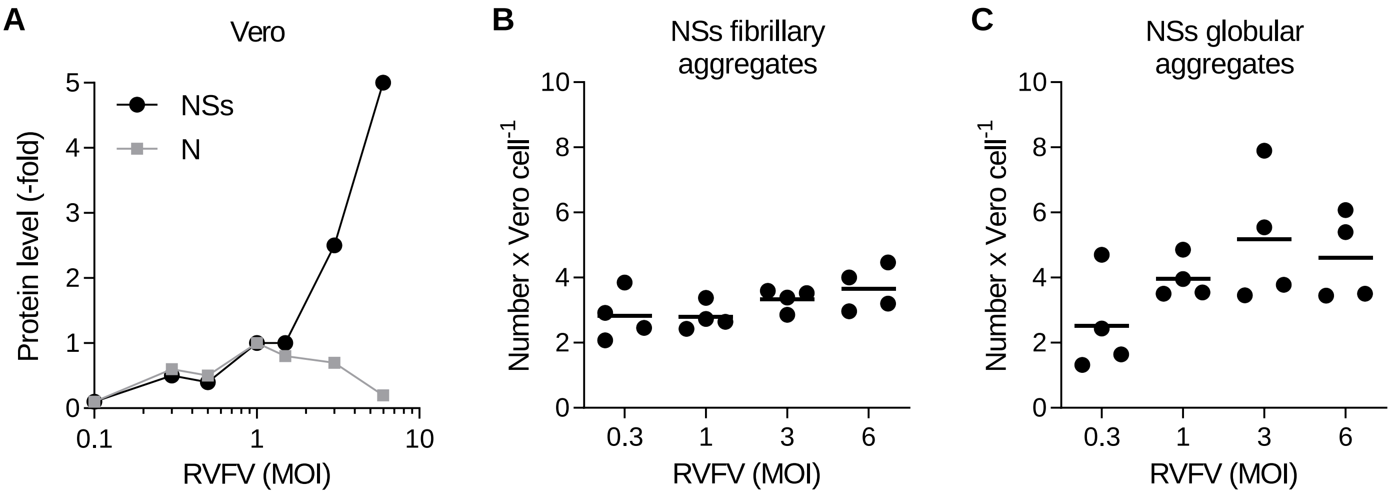

Supplementary Figure 7. The number of aggregates does not increase with high NSs expression. (A) Vero cells were infected at various MOIs of RVFV for 16 hr and assayed for the viral N and NSs proteins by WB as described in Figure 4A. The expression of both proteins was then semi-quantified. N and NSs levels are expressed as the fold-increase relative to their respective level in cells infected at a MOI of 1 and normalized to the level of tubulin. Results are representative of three independent experiments. (B and C) Vero cells were exposed to RVFV at MOIs ranging from 0.3 to 6, followed by immunofluorescence staining against N and NSs, and then, subjected to confocal microscopy imaging 16 hr later. Confocal Z-stack obtained were analyzed with the software ilastik as shown in Supplementary Figure 2. The number of fibrillary and globular aggregates is given per cell for the indicated MOIs. Points represent independent experiments (n = 4). Center line, mean.

Cell line	Species	Tissue	Nuclear filaments [detection post-infection (hr)]		
			MOI		
			0,1 <	1	> 2
A549	Human	Lung	ND	8 <	ND
BHK-21	Hamster	Kidney	ND	8 <	ND
H2.35	Murine	Liver	ND	ND	8 <
HEK-293T	Human	Kidney	ND	8 <	ND
HeLa	Human	Cervical carcinoma	8	6	6
HSAEC	Human	Lung	ND	ND	24 <
L-929	Murine	Fibroblast	8	6	5
OA4.K/S1	Sheep	Kidney	ND	ND	16 <
U-87 MG	Human	Neuron	ND	16 <	8 <
Vero	Monkey	Kidney	6	5	4-5

Supplementary Table 1. Cell lines supporting the formation of NSs filamentous aggregates. The table shows (i) a list of cells that support the formation of NSs filaments and (ii) the correlation between MOI and rapidity of nuclear NSs aggregation. ND, non-determined.

Primer (sense[a])	Sequence (5' -> 3')[b]	Purpose[c]
C1-F (Forw.)	*TATTCTATGGTTGGGCCCAGTAGTGTCTTTCTCATGCACC*	Mutagenesis NSs C39S/C40S
C1-R (Rev.)	*GGTGCATGAGAAAGACACTACTGGGCCCAACCATAGAATA*	Mutagenesis NSs C39S/C40S
C2-F (Forw.)	*CACTCTAGCAATGAGGAGTAGCAAGATCACCAATGA*	Mutagenesis NSs C149S
C2-R (Rev.)	*TCATTGGTGATCTTGCTACTCCTCATTGCTAGAGTG*	Mutagenesis NSs C149S
C3-F (Forw.)	*GAGGGATTGACCTGAGCCTGTTGCCAGGC*	Mutagenesis NSs C178S
C3-R (Rev.)	*GCCTGGCAACAGGCTCAGGTCAATCCCTC*	Mutagenesis NSs C178S
C4-F (Forw.)	*GTTGCTCACGTACAGAGCGTTCGGCTTCTGC*	Mutagenesis NSs C194S
C4-R (Rev.)	*GCAGAAGCCGAACGCTCTGTACGTGAGCAAC*	Mutagenesis NSs C194S
UTR-N-IR.F (Forw.)	AATCGTCTCTAGGT*ACACAAAGCTCCCTAGAGATACAAAC*	Engineering of the RVFV S segment encoding tc-NSs (3'UTR-N-IR5')
UTR-N-IR.R (Rev.)	AATCGTCTCCCGAC*CGTAACCCCAACTCCCCTTCC*	Engineering of the RVFV S segment encoding tc-NSs (3'UTR-N-IR5')
IR-tc-NSs-UTR.F (Forw.)	AATCGTCTCGGTCG*GGATTGGGGGGTGGGGGGTGGG*	Engineering of the RVFV S segment encoding tc-NSs (3'IR-tc-NSs-UTR5')
IR-tc-NSs-UTR.R1 (Rev.)	**ATGTTTCTCAATTGTTGCCCAGGCTGTTGCATGGAACCC***GATTACTTTCCTGTGATATC*	Engineering of the RVFV S segment encoding tc-NSs (3'IR-tc-NSs-UTR5'), 1st PCR
IR-tc-NSs-UTR.R2 (Rev.)	AATCGTCTCGGGG*ACACAAAGACCCCCTAGTGCTTATCAAGTATATC***ATGTTTCTCAATTGTTGCCC**	Engineering of the RVFV S segment encoding tc-NSs (3'IR-tc-NSs-UTR5'), 2nd PCR
IFN-β-mouse.F (Forw.)	CAGCTCCAAGAAAGGACGAAC	qRT-PCR-based analysis, *IFN-β* gene
IFN-β-mouse.R (Rev.)	GGCAGTGTAACTCTTCTGCAT	qRT-PCR-based analysis, *IFN-β* gene
HPRT1-mouse.F (Forw.)	CCTAAGATGAGCGCAAGTTGAA	qRT-PCR-based analysis, *HPRT1* gene
HPRT1-mouse.R (Rev.)	CCACAGGACTAGAACACCTGCTAA	qRT-PCR-based analysis, *HPRT1* gene

Supplementary Table 2. Names and sequences of the primers used in this study. [a] Forw., forward; Rev., reverse. [b] The virus RNA sequence that is targeted is in italics and the sequences introduced for cloning are in roman type. Underlined nucleotide sequences indicate a BsmBI restriction site. The tc peptide sequence used to tag the protein NSs in the N-terminal region appears in bold. [c] *HPRT1*, hypoxanthine phosphoribosyltransferase 1 gene; *IFN-β*, interferon-β gene; IR, intergenic region; N, RVFV nucleoprotein N; tc-NSs, RVFV tc-tagged NSs; tc, tetracysteine; qRT-PCR, real-time quantitative reverse transcription PCR; UTR, untranslated region.
