# Supplementary material for: NSs amyloid formation is associated with the virulence of Rift Valley fever virus in mice
Source: Nat Commun. 2020 Jul 1;11:3281. doi: 10.1038/s41467-020-17101-y (PMC7329897; doi:10.1038/s41467-020-17101-y)
Supplement: Supplementary file 3 — Reporting Summary [file 41467_2020_17101_MOESM3_ESM.pdf]

nature research

Corresponding author(s): Pierre-Yves Lozach

Last updated by author(s): Apr 9, 2020

Reporting Summary

Nature Research wishes to improve the reproducibility of the work that we publish. This form provides structure for consistency and transparency in reporting. For further information on Nature Research policies, see Authors & Referees and the Editorial Policy Checklist .

Statistics

For all statistical analyses, confirm that the following items are present in the figure legend, table legend, main text, or Methods section.

n/a	Confirmed	
☐	☒	The exact sample size (*n*) for each experimental group/condition, given as a discrete number and unit of measurement
☐	☒	A statement on whether measurements were taken from distinct samples or whether the same sample was measured repeatedly
☐	☒	The statistical test(s) used AND whether they are one- or two-sided<br>*Only common tests should be described solely by name; describe more complex techniques in the Methods section.*
☒	☐	A description of all covariates tested
☐	☒	A description of any assumptions or corrections, such as tests of normality and adjustment for multiple comparisons
☐	☒	A full description of the statistical parameters including central tendency (e.g. means) or other basic estimates (e.g. regression coefficient) AND variation (e.g. standard deviation) or associated estimates of uncertainty (e.g. confidence intervals)
☐	☒	For null hypothesis testing, the test statistic (e.g. *F*, *t*, *r*) with confidence intervals, effect sizes, degrees of freedom and *P* value noted<br>*Give P values as exact values whenever suitable.*
☒	☐	For Bayesian analysis, information on the choice of priors and Markov chain Monte Carlo settings
☒	☐	For hierarchical and complex designs, identification of the appropriate level for tests and full reporting of outcomes
☒	☐	Estimates of effect sizes (e.g. Cohen's *d*, Pearson's *r*), indicating how they were calculated

Our web collection on statistics for biologists contains articles on many of the points above.

Software and code

Policy information about availability of computer code

Data collection	Zen 2 (Zeiss), Imspector 16.1.7098 (Abberior Instruments), LAS X (Leica), Volocity 6.3 (PerkinElmer), NIS Elements 4 (Nikon)
Data analysis	ImageJ 1.52p (NIH, BSD license), Imaris 8.0.2 (Bitplane), Icy 1.9.10.0 (Pasteur Institute, GPLv3 license), Imspector 16.1.7098 (Abberior Instruments), Ilastik 1.3.3 (University Heidelberg, GPL2 license), Prism 8.4.1 (GraphPad Software)

For manuscripts utilizing custom algorithms or software that are central to the research but not yet described in published literature, software must be made available to editors/reviewers. We strongly encourage code deposition in a community repository (e.g. GitHub). See the Nature Research guidelines for submitting code & software for further information.

Data

Policy information about availability of data

All manuscripts must include a data availability statement. This statement should provide the following information, where applicable:

- Accession codes, unique identifiers, or web links for publicly available datasets
- A list of figures that have associated raw data
- A description of any restrictions on data availability

Authors can confirm that all relevant data are included in the paper and the supplementary information files. The material and data that support the findings of this study are also available from the corresponding author upon reasonable request.

Field-specific reporting

Please select the one below that is the best fit for your research. If you are not sure, read the appropriate sections before making your selection.

Life sciences study design

All studies must disclose on these points even when the disclosure is negative.

Sample size	No statistical methods were used to determine sample size. We determined the sample size so that the size was sufficient to reach the statistical significance when different conditions were assessed. Sample sizes and statistical tests used in the study are clearly written in the methods and figure legends.
Data exclusions	No data were excluded from the analyses.
Replication	Microscopy and blot images are representative of at least three individual experiments. All image quantifications are shown as the mean or median of replicates acquired in different microscopy fields. Results from virus titration and qRT-PCR are representative of at least two independent experiments and are given as the mean of tri- or quadruplicates. Replication is clearly written in the methods and figure legends.
Randomization	No specific randomization approach was used. Though the study only involves virus clones and cell and murine clonal lines, viruses and cells were however pseudo-randomly assigned per condition, and animals were selected based on their age and sex.
Blinding	This is not relevant to our field for obvious biosafety reasons. The virus studied in this investigation requires a biosafety level 3 laboratory, and investigators needed to know what they were working with. The investigators were therefore not blinded during data collection or analysis.

Reporting for specific materials, systems and methods

We require information from authors about some types of materials, experimental systems and methods used in many studies. Here, indicate whether each material, system or method listed is relevant to your study. If you are not sure if a list item applies to your research, read the appropriate section before selecting a response.

Materials & experimental systems

n/a	Involved in the study	
☐	☒	Antibodies
☐	☒	Eukaryotic cell lines
☒	☐	Palaeontology
☐	☒	Animals and other organisms
☒	☐	Human research participants
☒	☐	Clinical data

Methods

n/a	Involved in the study	
☒	☐	ChIP-seq
☒	☐	Flow cytometry
☒	☐	MRI-based neuroimaging

Antibodies

Antibodies used	The mouse monoclonal antibody 1D8 (Saluzzo JF et al., 1989, Res. Virol.) was used to detect the RVFV nucleoprotein N while the rabbit polyclonal antibodies SE2323 (Billecocq et al., 2000, J. Gen. Virol.) and 2284 (Le May et al., 2004, Cell) were used against the RVFV proteins N and NSs, respectively. Commercial antibodies were used to detect actin (A2228, Sigma Aldrich, clone AC74, lot number 085M4754V), tubulin (T5168, Sigma Aldrich, clone B512, lot number 038M4813V), and PKR (18244-1-AP, Proteintech, lot number 00045302).<br>The following secondary antibodies were used in this study: goat anti-mouse Alexa Fluor (AF) 488-conjugated Abs (Thermo Fisher Scientific, #A-11001), goat anti-rabbit AF568-conjugated Abs (Thermo Fisher Scientific, #A-11011), goat anti-rabbit AF555-conjugated Abs (Thermo Fisher Scientific, #A-21428), goat anti-rabbit AF647-conjugated Abs (Thermo Fisher Scientific, #A-21244), alpaca anti-rabbit alkaline phosphatase-conjugated Abs (Jackson ImmunoResearch Laboratories, 611-055-215), goat anti-rabbit horseradish peroxidase-conjugated Abs (Vector Laboratories, PI-1000), and horse anti-mouse horseradish peroxidase-conjugated Abs (Vector Laboratories, PI-2000).
Validation	The 1D8 antibody was previously validated for the RVFV nucleoprotein N detection by immunoblot in Le May et al., 2005, J. Virol. [79(18):11974-80].<br>The SE2323 antibody was previously validated for the RVFV nucleoprotein N detection by immunofluorescence detection in Billecocq et al., 2000, J. Gen. Virol. [81(Pt 9):2161-66].<br>The 2284 antibody was previously validated for the RVFV non-structural protein NSs detection by immunofluorescence and immunoblot detection in Le May et al., 2004, Cell [116(4):541-50].<br>From the manufacturer's website (https://www.sigmaaldrich.com/catalog/product/sigma/a2228?lang=de&region=DE), the anti-actin antibody AC74 (A2228, Sigma Aldrich) reacts with beta actin from a wide range of species and is validated for applications such as immunochemistry, microarray, and western blot.<br>From the manufacturer's website (https://www.sigmaaldrich.com/catalog/product/sigma/t5168?lang=de&region=DE), the anti-tubulin antibody B512 (T5168, Sigma Aldrich) reacts with alpha tubulin from human, monkey, and other species. It is validated for applications such as immunofluorescence and western blot.<br>From the manufacturer's website (https://www.ptglab.com/Products/EIF2AK2-Antibody-18244-1-AP.htm), the anti-PKR antibody (18244-1-AP, Proteintech) reacts with human PKR and is validated for applications such as western blot and immunofluorescence (Borghese et al., 2019, J. Virol.).

Eukaryotic cell lines

Policy information about cell lines

Cell line source(s)	The baby hamster kidney fibroblastic cells BHK/T7-9 expressing T7 polymerase were obtained from Makoto Sugiyama (Gifu University, Japan). The murine fibroblastic cells (L-929, CCL-1) were purchased from ATCC. All the other cell lines were originally purchased from ATCC but obtained from different research laboratories. In the details, the human kidney epithelial cells (HEK-293T), the human epithelial lung cells (A549), and the monkey kidney epithelial cells (Vero) were a generous gift from Prof. Ari Helenius (ETH Zurich, Switzerland). The human brain epithelial cells (U-87 MG) were obtained from Prof. Barbara Müller (University Hospital Heidelberg, Germany).
Authentication	Cells were visually authenticated by their morphology but not genetically confirmed.
Mycoplasma contamination	All cell lines were tested negative by PCR for mycoplasma contamination.
Commonly misidentified lines (See ICLAC register)	No misidentified cell lines were used in this study.

Animals and other organisms

Policy information about studies involving animals; ARRIVE guidelines recommended for reporting animal research

Laboratory animals	Laboratory animals involved nine-week-old BALB/cByJ male mice. Animals were bred in an SPF animal house and infected in a BSL-3 animal facility. They were maintained at 22+/- 2°C and 55 +/- 15% humidity under a 14:10 light-dark cycle. They were fed standard chow ad libitum and received autoclaved demineralized water.
Wild animals	The study did not involve wild animals.
Field-collected samples	The study did not involve samples collected from the field.
Ethics oversight	Mice experiments were conducted according to the French and European regulations on animal care and protection (EC Directive 2010/63/UE and French Law 2013-118 issued on 2013/2/1). The experimental protocol was approved by the Institut Pasteur Ethics Committee under #2016-0013 and authorized by the French Ministry of Research under #06463.

Note that full information on the approval of the study protocol must also be provided in the manuscript.
