# Supplementary material for: NSs amyloid formation is associated with the virulence of Rift Valley fever virus in mice
Source: Nat Commun. 2020 Jul 1;11:3281. doi: 10.1038/s41467-020-17101-y (PMC7329897; doi:10.1038/s41467-020-17101-y)
Supplement: Supplementary file 4 — Description of Additional Supplementary Files [file 41467_2020_17101_MOESM4_ESM.pdf]

Description of Additional Supplementary Files

File Name: Supplementary Movie 1
Description: Dynamics of NSs aggregation. RVFV tc-NSs (MOI ~5) was added to Vero cells for 3 hr. Input virus was then exchanged against phenol red-free medium containing the ReAsH dye (200 nM), and video recording started. Cells were visualized by wide-field microscopy at 37°C and images taken at 1/300 Hz in one plane for 23 hr. Video is displayed at 15 Hz. NSs aggregates (red) are seen being assembled.

File Name: Supplementary Movie 2
Description: 3D-reconstruction of infected mouse brain tissues expressing N. BALB/c mice were inoculated intraperitoneally with 100 plaque-forming units (pfu) of RVFV. When the first disease symptoms appeared, animals were sacrificed and brains collected, fixed, and subjected to immunostaining against N (red) and labeling of nuclei with Hoechst (blue). 3D-reconstruction from Z-stacks obtained by high-speed confocal microscopy and the IMARIS software is shown.

File Name: Supplementary Movie 3
Description: 3D-reconstruction of infected mouse brain tissues expressing NSs. BALB/c mice were inoculated intraperitoneally with 100 pfu of RVFV. When the first disease symptoms appeared, animals were sacrificed and brains collected, fixed, and subjected to immunostaining against NSs (red) and labeling of nuclei with Hoechst (blue). 3D-reconstruction from Z-stacks obtained by high-speed confocal microscopy and the IMARIS software is shown.
